# The impairing effects of mental fatigue on response inhibition: An ERP study

**DOI:** 10.1371/journal.pone.0198206

**Published:** 2018-06-01

**Authors:** Zizheng Guo, Ruiya Chen, Xian Liu, Guozhen Zhao, Yan Zheng, Mingliang Gong, Jun Zhang

**Affiliations:** 1 CAS Key Laboratory of Behavioral Science, Institute of Psychology, Beijing, China; 2 School of Transportation and Logistics, Southwest Jiaotong University, Chengdu, China; 3 National United Engineering Laboratory of Integrated and Intelligent Transportation, Southwest Jiaotong University, Chengdu, China; 4 Department of Psychology, University of Chinese Academy of Sciences, Beijing, China; 5 Department of Psychology, Miami University, Oxford, United States of America; University of Pennsylvania, UNITED STATES

## Abstract

Mental fatigue is one of the main reasons for the decline of response inhibition. This study aimed to explore the impairing influence of mental fatigue on a driver’s response inhibition. The effects of mental fatigue on response inhibition were assessed by comparing brain activity and behavioral indices when performing a Go/NoGo task before and after a 90-min fatigue manipulation task. Participants in the driving group performed a simulated driving task, while individuals in the control group spent the same time watching movies. We found that participants in the driving group reported higher levels of mental fatigue and had a higher percentage of eye closure and larger lateral deviations from their lane positions, which indicated there was effective manipulation of mental fatigue through a prolonged simulated driving task. After manipulation of mental fatigue, we observed increased reaction time and miss rates, delayed NoGo-N2 latency and Go-P3 latency, and decreased NoGo-P3 amplitude, which indicated that mental fatigue may slow down the speed of the inhibition process, delay the evaluation of visual stimuli and reduce the availability of attentional resources. These findings revealed the underlying neurological mechanisms of how mental fatigue impaired response inhibition.

## Introduction

Response inhibition, which is a core component of executive function[1], refers to the ability to inhibit inappropriate or irrelevant responses [2–4]. Response inhibition can be roughly divided into two processes: the monitoring of conflict and the evaluation of stimuli or resource allocation [5]. Many daily activities are associated with response inhibition. For example, when an emergent road event occurs (e.g., a lead vehicle suddenly stops), the driver has to brake sharply, and failure to do so may lead to a catastrophic accident [6–9]. Specifically, this operation consists of two processes that include inhibiting the process of stepping on the gas and initiating the process of slamming on the brakes.

However, an individual cannot maintain response inhibition at a high level all the time. Mental fatigue is one of the main reasons for the decline in response inhibition. Mental fatigue is a subjective feeling of tiredness or decreased cognitive ability that people experience during or after a prolonged period of cognitive activity that requires sustained mental efficiency [10–12]. Some previous studies have examined the detrimental effects of mental fatigue on response inhibition by employing Go/NoGo tasks that are generally used to assess the response inhibition process [13]. The Go/NoGo task requires participants to make a response to target stimuli (Go) but not to deviant stimuli (NoGo). The reaction time for the correct trials, the miss rate for the Go stimuli and the false alarm rate for the NoGo stimulus have been used as the behavioral performance indicators in Go/NoGo tasks. These three indicators increase as a larger amount of time is spent on tasks [14–16], which indicates there is an adverse influence of mental fatigue on response inhibition.

Although behavioral indices can reflect the effects of mental fatigue on response inhibition, merely relying on behavioral measures cannot explain how mental fatigue affects the cognitive process of response inhibition. Event-related potential (ERP), due to its high temporal resolution, has been widely adopted to explore cognitive processes [17, 18]. Three ERP components, including NoGo-N2, Go- and NoGo-P3, have been shown to be sensitive to diverse phases of response inhibition in Go/NoGo tasks [19, 20]. The first component of NoGo-N2 is enhanced by post-NoGo stimuli onset and represents an earlier process of response inhibition, i.e., the monitoring of conflict [21, 22]. The latency of the NoGo-N2 component reflects the speed of the monitoring of conflict, and the amplitude of the NoGo-N2 component represents the intensity of conflicts between trials [23, 24]. Go-P3 and NoGo-P3 belong to the same component, P3, and are elicited by post-Go and post-NoGo stimuli onset, respectively; they represent a later process of response inhibition, i.e., the evaluation of stimuli or resource allocation [25, 26]. The latency of the P3 component reflects the stimulus-evaluation speed, and the amplitude of the P3 component shows the amount of cognitive resources [26–29].

Previous studies have explored the effects of mental fatigue on response inhibition by employing ERP. Kato, Endo (14) reported that the latency of NoGo-N2, Go- and NoGo-P3 were delayed, and the amplitude of the NoGo-P3 decreased due to mental fatigue caused by a large amount of time spent on a Go/NoGo task, but the amplitudes of NoGo-N2 and Go-P3 were not affected. Their results revealed that mental fatigue tended to slow down the time course of response inhibition. In contrast, Falkenstein, Hoormann (13) designed the same Go/NoGo task to induce mental fatigue but found that neither N2 nor P3 components were affected by mental fatigue, which reflected a robust inhibitory process against mental fatigue. Taken together, the effects of mental fatigue on response inhibition are inconsistent and have yet to be clarified.

Traditional investigations of the influence of mental fatigue on response inhibition use a prolonged period of classical psychological cognitive tasks to induce mental fatigue, such as the Flanker task [10], Simon task [30] and Go/NoGo task [14]. Due to a relatively simple “stimuli-response” mechanism, a prolonged period of these cognitive tasks may induce mental fatigue confounded with an emotional aversion to boredom and/or monotony [31]. Therefore, experimental tasks with higher ecological validity are needed to induce a pure and single mental state of fatigue [32]. In addition, the previous investigations of the influence of mental fatigue on response inhibition are the lack of control group. The control group that does not receive such a prolonged period of cognitive tasks serves as a benchmark to evaluate how the mental fatigue is induced. Without the control group, for example, learning effects may have an impact on the accuracy of experimental results. In other words, the more times a task has been performed, the shorter the response time is and the fewer cognitive resources are required for each subsequent iteration. Therefore, a control group must be added in future investigations to exclude the potential influences of irrelevant variables and enhance the reliability of experimental results.

In the current study, a simulated driving task was adopted to induce mental fatigue. Compared to traditional cognitive tasks, driver fatigue is the natural result of physical and/or mental exertion that impairs driving performance and nobody is immune to the effects of driver fatigue [33, 34]. The aim of this study was to explore the influence of mental fatigue on a driver’s response inhibition in a Go/NoGo task. The effects of mental fatigue on response inhibition were assessed by comparing the ERPs and behavioral indices when performing a Go/NoGo task before and after a 90-min simulated driving task. We hypothesized that mental fatigue would result in a deterioration in response inhibition, which would be reflected through increases in the reaction time, miss rate of Go stimuli, and error rate of NoGo stimuli. Furthermore, we expected that mental fatigue would impair the process of Go stimuli and the inhibitory process of NoGo stimuli, which would be reflected by a decreased P3 amplitude and an increased N2/P3 latency.

## Materials and methods

### Participants

Sixty-six Chinese undergraduate or graduate students responded to the recruitment advertisements. We excluded volunteers with a history of neurological or psychiatric illness and individuals taking specific medications that influence the central nervous system. Finally, sixty of the volunteers (30 females, 30 males) were selected, and their ages ranged from 18 to 27 years old (average = 23.25, SD = 2.5 years). All participants held a valid driver license. They were right-handed and had normal or corrected-to-normal vision. They were required to avoid alcohol and caffeine on the day of experiment as well as to refrain from eating or exercising 2 hours before the study. All participants provided written informed consent and were paid for their participation. This study was approved by the Ethics Committee of Human Experimentation at the Institute of Psychology. All research activities were performed according to relevant guidelines and regulations outlined in the approved protocol.

### Experimental design

A 2 × 2 mixed design was used to examine group differences in the ERPs and behavioral indices when performing a Go/NoGo task before and after a fatigue manipulation task. The group served as a between-subjects variable with two levels: a driving group and a control group. Participants in the driving group (15 females and 15 males, age: 23.0 ± 2.4 years, driving experience: 7766 ± 1774 kms) completed a 90-min simulated driving task. Participants in the control group (15 females and 15 males, age: 23.5 ± 2.6 years, driving experience: 7600 ± 1830 kms) spent 90 mins on watching movies. The session served as a within-subjects variable with two levels: pre-test (participants completed a Go/NoGo task before fatigue manipulation task) and post-test (participants completed a Go/NoGo task after fatigue manipulation task).

### Experimental tasks

#### Go/NoGo task

**Fig 1:**
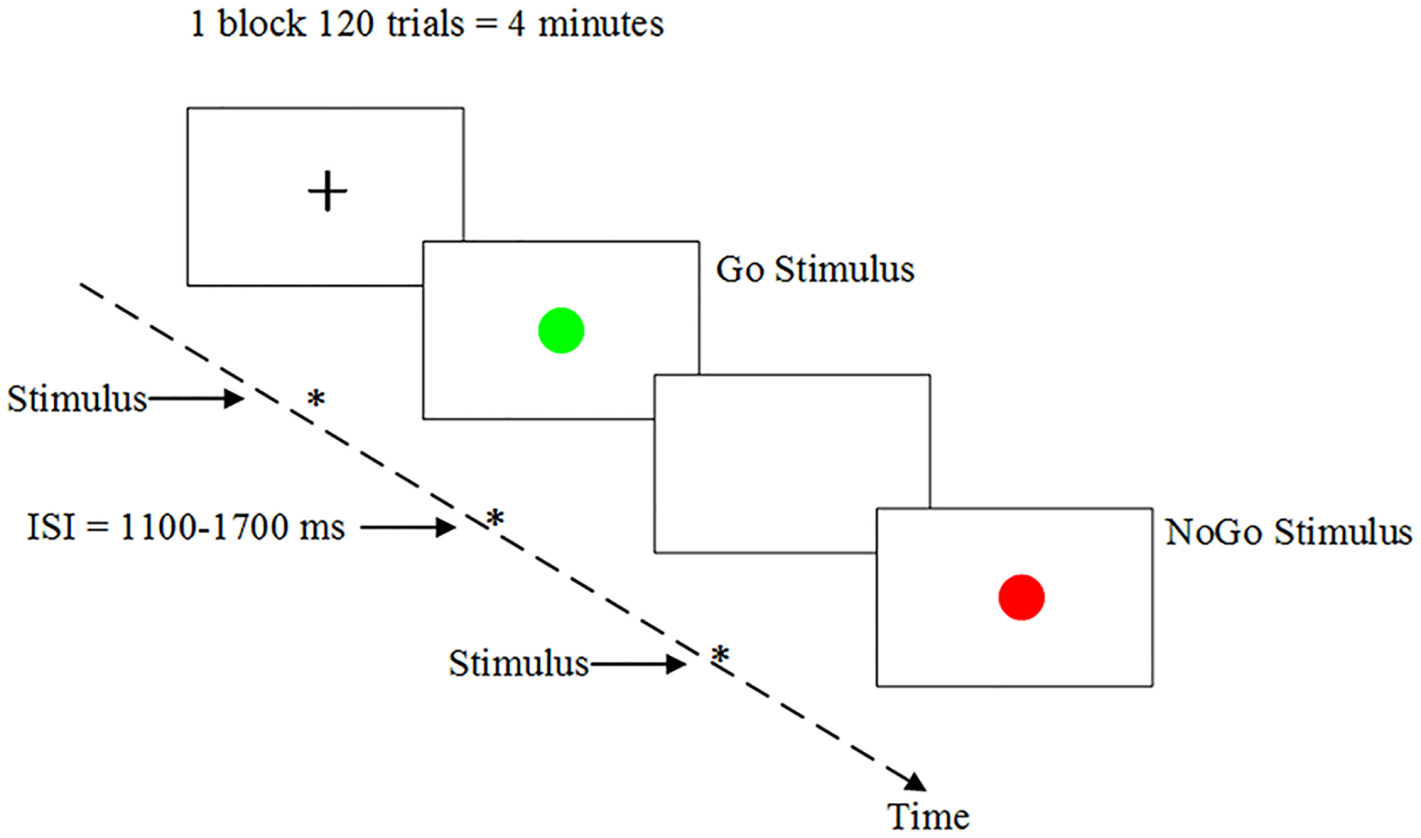
The timing scheme for the Go/NoGo task.

In the Go/NoGo task, the stimulus was either a green dot (Go stimulus) or red dot (NoGo stimulus). In each trial, a dot (green or red) was presented in the center of the screen with a visual angle of approximately 2.5°. Stimuli were presented for 200 ms with a random stimulus interval of 1100–1700 ms, as illustrated in Fig 1. The numbers of Go and NoGo stimuli were 384 (80%) and 96 (20%), respectively. The sequences of these stimuli were pseudo-random to avoid the appearance of several NoGo stimuli in a row.

#### Fatigue manipulation task

**Fig 2:**
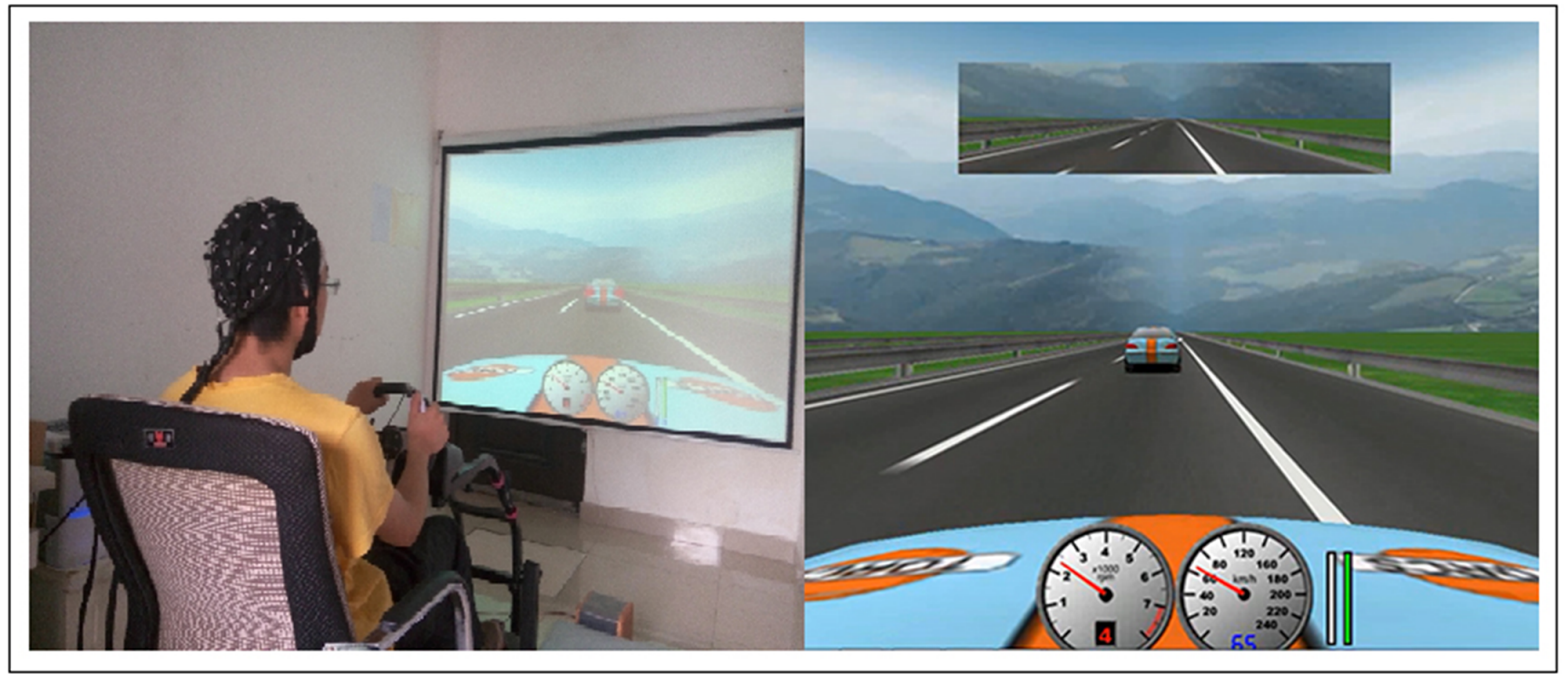
Driving simulator (left) and TORCS-based [35] driving scenarios (right). The participant in Fig 2 has given written informed consent (as outlined in PLOS consent form) to publish these case details.

In the driving task, a fixed-base driving simulator with a force-feedback steering wheel combined with open source driving simulation software (The Open Racing Car Simulator, TORCS [35]) was adopted. A tachometer, a speedometer, and an inside mirror were displayed at 1024 × 768 resolution. The driving scenario was a simulated expressway (right-hand traffic) with two lanes in each direction. The road was a flat surface curve consisting of a series of straight segments and horizontal curves. An EPSON projector (EPSON, TW495, China) was used to present the driving scenarios. Participants in the driving group were asked to follow a lead vehicle with a speed that varied randomly from 55 km/h to 65 km/h and maintain the minimum safe distance. Overtaking the lead vehicle and changing lanes were not permitted. The lead vehicle was programmed to brake occasionally, making the brake light on, and participants were instructed to press the key as quickly as possible when they saw the brake light go on. Each brake event was regarded as a trial, and there were 180 trials with a random interval of 25–35 s between two consecutive trials. The simulated driving task lasted for 90 mins without a rest. The simulated driving interface was shown in Fig 2. Participants in the control group were asked to watch movies we provided for the same period of time (90 mins). The movies named Frozen Planet which consists of a set of documentary series with seven episodes.

### Experimental procedure

**Fig 3:**
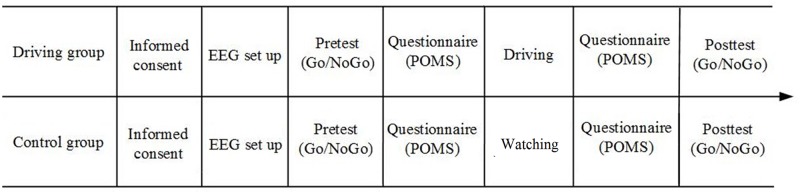
Flow diagram of the experiment.

The experimental procedure is illustrated in Fig 3. After arriving at the lab at 14:30, the participants handed over their watches and cellphones, had a 10-min rest and were then provided informed consent. They were seated in a dimly lit, electrically shielded room, and each participant put on an EEG cap with the assistance of two experimenters. The Go/NoGo task consisted of a preliminary session and an experimental session. In the preliminary session, the participants completed 20 trials. The experimental session consisted of 4 blocks of 480 trials. Each block was 4 minutes and was followed by a 3-min break and the fatigue questionnaire. After they filled out the questionnaire, a 90-min simulated driving task (driving group) or watching movies task (control group) was implemented to induce mental fatigue, which was followed by repeating the same fatigue questionnaire. Both groups of participants went through the same Go/NoGo task in the first session. The whole experiment lasted no more than 2.5 hours.

Subjective mental fatigue was assessed with the fatigue subscale of Profile of Mood States Short Form (POMS-SF) in Chinese [36]. POMS-SF seems to be the most valid and reliable instrument to assess an individual’s momentary mood states. The fatigue subscale consists of 5 adjectives describing fatigue, including worn out, fatigued, exhausted, sluggish and weary. Participants scored every adjective from 0 (not at all) to 4 (extremely), and the total score was calculated and adopted [10, 36]. The pupillary diameter was continuously recorded by the SMI HED eye tracker at 120 Hz sampling rate.

### EEG acquisition

Electroencephalogram (EEG) data were continuously recorded from 64 active electrodes attached to an electrode elastic cap (Neuroscan Inc., Charlotte, NC). Electrode positions included the standard International 10–20 system locations and intermediate sites. Vertical and horizontal electrooculograms (EOG) were recorded from electrodes fixed above and below the left eye and placed 10 mm from the outer canthi of both eyes, respectively. The left mastoid was used as an online reference for all channels. The EEG data were digitized at 500 Hz. All electrode impedances were kept below 5 kΩ.

We used Neuroscan software 4.5 to process EEG data. The EEG data were amplified by the FIR filter with a 0.1–30 Hz bandpass, the Zero Phase Shift filtering mode was adopted and the decay rate was 24 dB/oct. Filtered EEG data were segmented into epochs with artifact voltages exceeding the threshold that varied among the participant population [37]. Ocular artifacts were removed from the EEG raw data using a regression procedure implemented in the Neuroscan software. Clean EEG data were re-referenced to the average of the left mastoids and right mastoids. After visual inspection, the EEG data were segmented into epochs that ranged from -200 ms to 600 ms after the onset of the red/green dots. The single-trial epoch was then baseline corrected and averaged to form a grand average ERP. The average valid trial numbers of Go stimuli were 361 and 363 in the driving group (before and after the simulated driving task) and were 369 and 367 in the control group (before and after watching movies). The average valid trial numbers of NoGo stimuli were 89 and 87 in both driving and control groups (pre- and post-test). We analyzed the ERPs that were stimulus-locked to the onset of the green dots (Go stimulus) and red dots (NoGo stimulus). The P3 component in both Go and NoGo trials and N2 component in NoGo trials were analyzed. We measured peak amplitudes (from baseline) and latencies (from stimuli onset) of the P3 component over three midline sites (Fz, Cz, and Pz) where they were maximal positive values from a 350–600 ms time window. The NoGo-N2 amplitudes and latencies at Fz and Cz sites were measured as peak values of the negative component at 200–350 ms poststimulus intervals [14]. Peak analyses were performed on individual data for each trial.

### Data analysis

Three behavioral indices were calculated to indicate performance in a Go/NoGo tasks: the reaction time (RT) for correct trials, the miss rate for Go stimuli, and the false alarm rate for the NoGo stimuli. Subjective ratings of mental fatigue before and after fatigue manipulation task and the percentage of eye closure (PERCLOS) [38] and standard deviation of the lane position (SDLP) [39] in the first and last 30 mins of the simulated driving task were also calculated to reflect the level of mental fatigue. The formulas of reaction time, miss rate, PERCLOS and SDLP are shown in the following:

$$RT=\frac{\sum_{k=1}^{n}RT_{k}}{n_{1}}$$ (1)

Here, *RT* was the mean value of the reaction time on Go stimuli, *RT*_*k*_ is the reaction time on Go stimuli at number *k*, *n*_*1*_ is the amount of Go stimuli that corresponding to correctly.

$$P_{miss}=\frac{n_{m}}{n_{2}}\times 100\%$$ (2)

Here, *P*_*miss*_ was the miss rate on Go stimuli, *n*_*m*_ was the amount of missed Go stimuli, *n*_*2*_ was the amount of all the Go stimuli.

$$P_{false}=\frac{n_{f}}{n_{3}}\times 100\%$$ (3)

Here, *P*_*false*_ was the false alarm rate, *n*_*f*_ was the amount of the false alarmed NoGo stimuli, *n* was the amount of all the NoGo stimuli.

$$PERCLOS=\frac{A}{N_{2}}\times 100\text{\%}$$ (4)

Here, *A* was the sample size of normalized pupillary diameters which were less than or equal to 20%, *N*_*2*_ was the sample size of all the pupillary diameter.

$$SDLP=\sqrt{\frac{1}{N_{1}}\sum_{i=1}^{N_{1}}{\left(LP_{i}-LP\_mean\right)}^{2}}$$ (5)

Here, *i* was the time, *LP*_*i*_ was the lane position at time *i*, *LP_mean* was the mean value of the lane position, *N*_*1*_ was the sample size of the lane position.

Paired sample *t*-tests were adopted to examine differences in the PERCLOS and SDLP between the first 30 mins and the last 30 mins of the 90-min simulated driving task. A repeated measures analysis of variance (ANOVA) was performed on the subjective ratings of mental fatigue, behavioral performance and ERPs with the group as a between-subjects factor and the session as a within-subjects factor. Significant session × group interactions were followed-up with a simple effect analysis to assess the effects that the session has on the dependent variables for each group. The Pearson correlation analyses were performed between ERP changes at the Fz site (differences in Go-P3, NoGo-P3, and NoGo-N2 amplitudes and latencies before and after the simulated driving task in the driving group) and mental fatigue indices (differences in POMS, SDLP, and PERCLOS before and after the simulated driving task in the driving group) and behavior performance decrease (differences in RT, miss rate and false alarm before and after the simulated driving task in the driving group).

## Results

### Manipulation of mental fatigue

**Fig 4:**
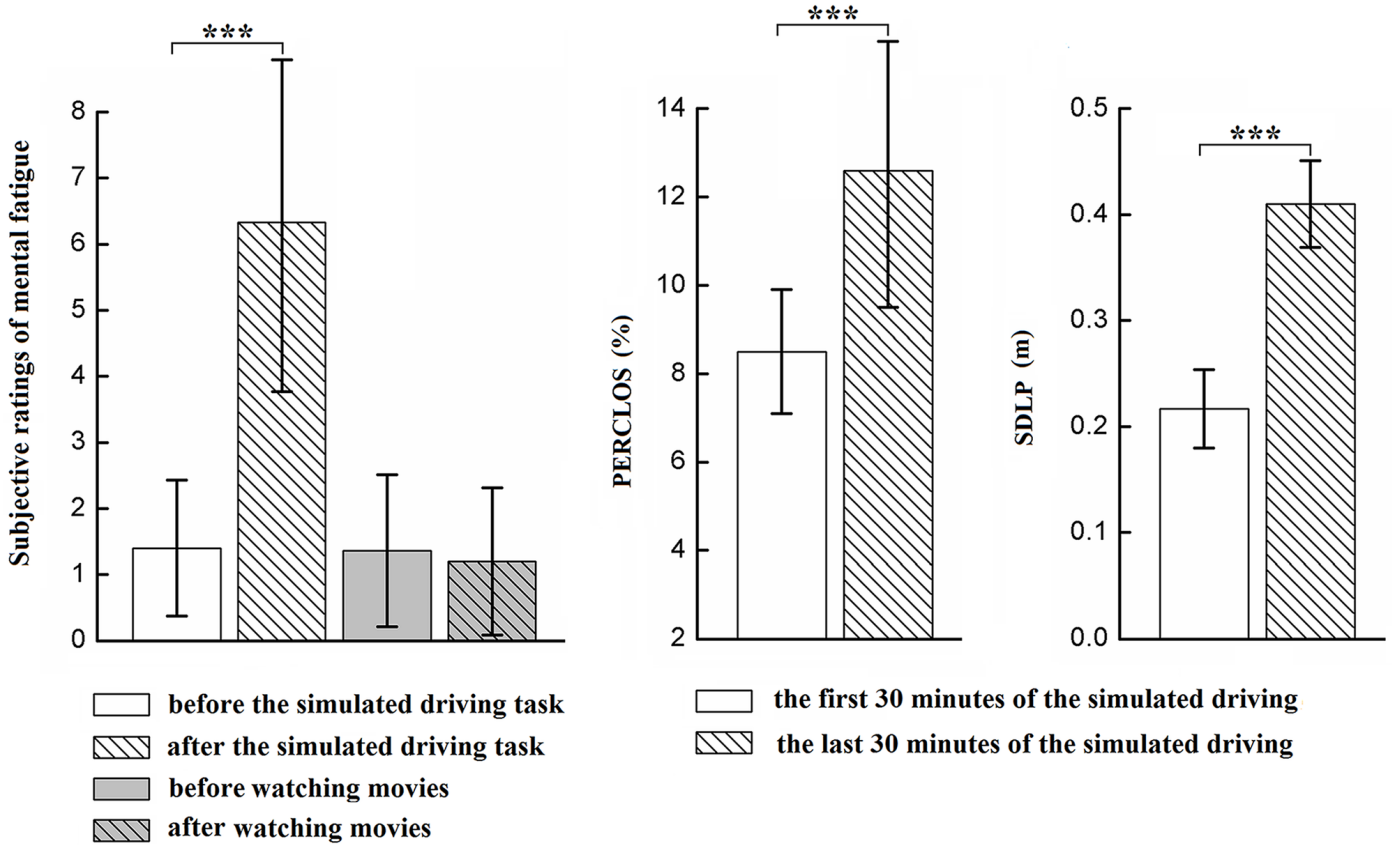
A significant session × group interaction for the subjective ratings of mental fatigue (left), significant differences in the percentage of eye closure (middle) and standard deviation of the lane position (right) between the first 30-min and the last 30-min of the simulated driving task in the driving group. Error bar indicates ±1 standard deviation. *p < 0.05, **p < 0.01, ***p < 0.001.

The interaction between session and group was significant for the subjective ratings of mental fatigue (*F*(1, 58) = 85.689, *p* < 0.001, λ = 0.404) (see Fig 4). Simple effect analysis indicated that participants in the driving group reported higher levels of mental fatigue after a 90-min simulated driving task (M = 6.20, SD = 2.52, *p* < 0.001), but there was no significant difference before and after watching movies in the control group. The main effect of the session was significant for the subjective ratings of mental fatigue (*F*(1, 53) = 74.353, *p* < 0.001, λ = 0.438).

Moreover, paired-sample t-tests showed significant differences in the PERCLOS (*t*(29) = -7.226, *p* < 0.001) and the SDLP (*t*(29) = -17.654, *p* < 0.001) between the first 30 mins and the last 30 mins of the 90-min simulated driving task (see Fig 4). Participants in the driving group had a higher percentage of eye closure (M = 12.6%, SD = 3.1%) and larger lateral deviations from their lane positions (M = 0.41 m, SD = 0.041 m) in the last 30 mins of the simulated driving task. These results revealed that a 90-min simulated driving task successfully induced a higher level of mental fatigue than a 90-min watching movies task.

### Behavioral performance in Go/NoGo tasks

**Fig 5:**
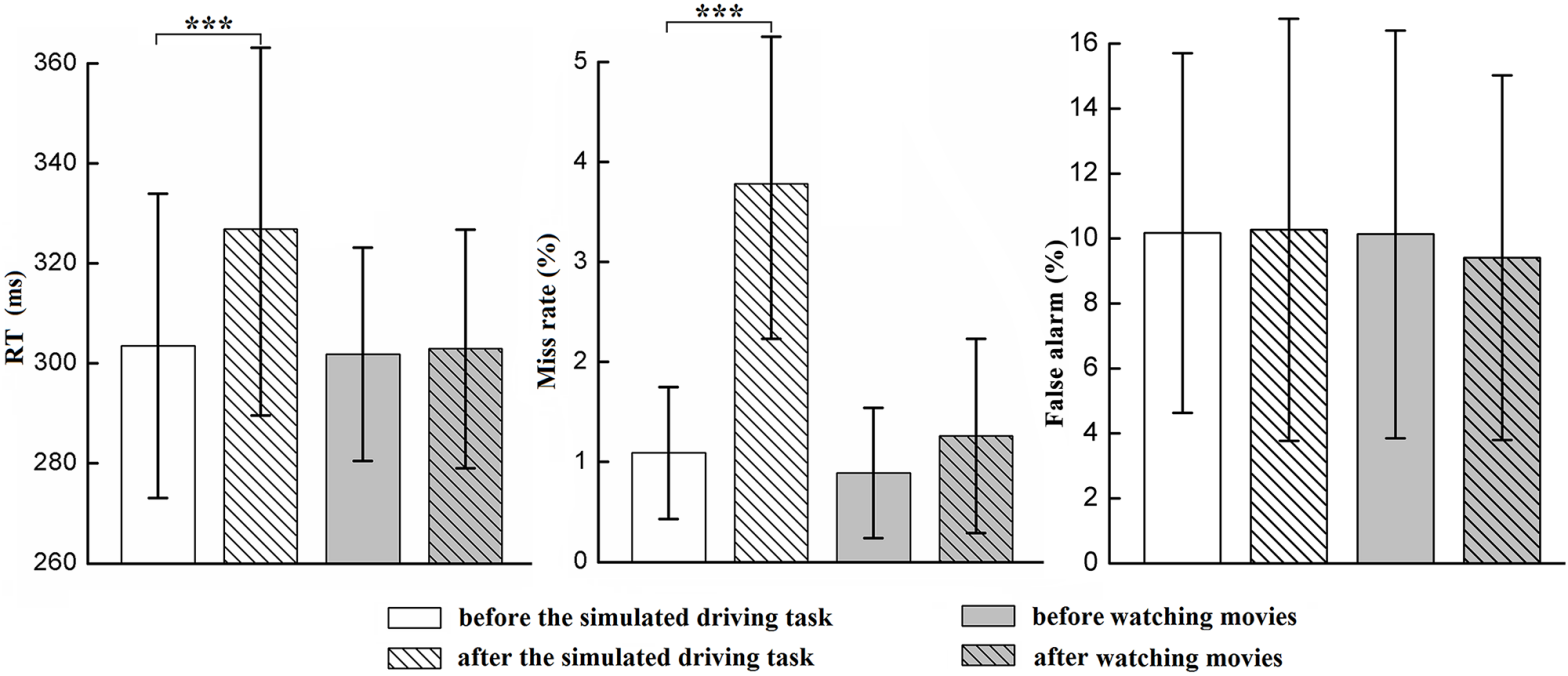
Significant session × group interactions for reaction time (left) and the percentage of misses for the Go trials (middle) and no significant differences in the percentage of false alarms for the NoGo trials (right) between the driving and control group or before and after manipulation of mental fatigue. Error bar indicates ±1 standard deviation. *p < 0.05, **p < 0.01, ***p < 0.001.

There were significant session × group interactions for the measures of reaction time (*F*(1, 58) = 23.656, *p* < 0.001, λ = 0.710) and miss rate (*F*(1, 58) = 57.744, *p* < 0.001, λ = 0.501) (see Fig 5). Simple effect analyses showed that participants in the driving group had longer reaction times (M = 326.87 ms, SD = 37.32 ms) and a higher percentage of misses for the Go trials (M = 3.78%, SD = 1.55%) after a 90-min simulated driving task (*ps* < 0.001). In contrast, both reaction time and miss rate remained at the same level before and after manipulation of mental fatigue. Additionally, both reaction time (*F*(1, 58) = 31.482, *p* < 0.001, λ = 0.648) and miss rate (*F*(1, 58) = 98.957, *p* < 0.001, λ = 0.370) significantly increased after manipulation of mental fatigue, which indicated there was an adverse influence of mental fatigue on response inhibition. However, the percentage of false alarms on the NoGo trials did not show any significant difference between the driving and control group or before and after manipulation of mental fatigue.

### Event-related potentials

#### Go condition

**Fig 6:**
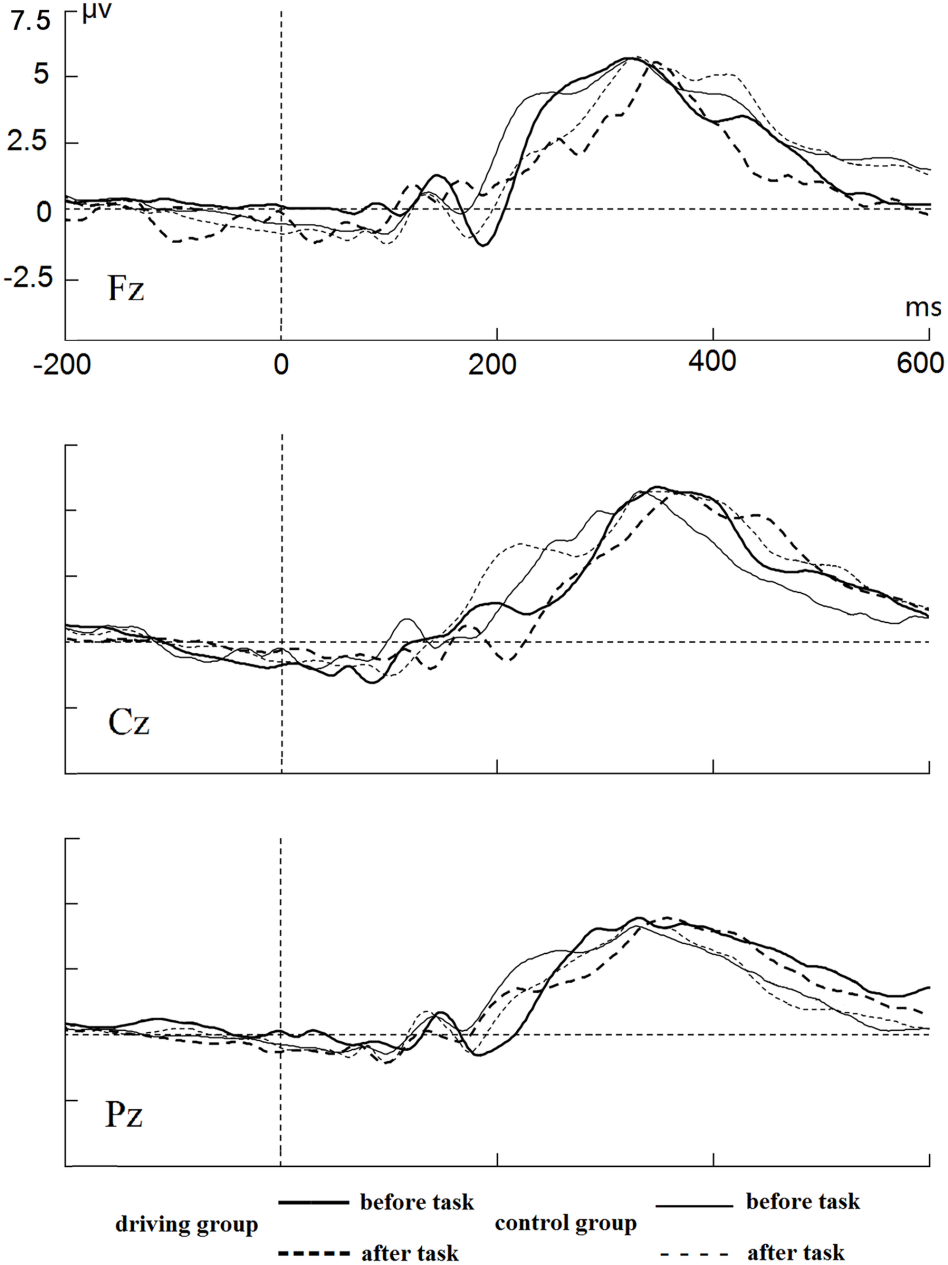
Average ERPs at Fz, Cz and Pz sites for Go trails.

Fig 6 shows the average ERP at the Fz, Cz and Pz sites in the Go trials. Only the P3 component was successfully elicited in the Go trials. There were significant interactions between session and group for the Go-P3 latency at Fz (*F*(1, 58) = 6.734, *p* < 0.05, λ = 0.896), Cz (*F*(1, 58) = 11.750, *p* < 0.01, λ = 0.832), and Pz (*F*(1, 58) = 8.119, *p* < 0.01, λ = 0.877). Simple effect analyses showed that the Go-P3 latency significantly increased after manipulation of mental fatigue for the driving group (*ps* < 0.001) at Fz (M = 340.00 ms, SD = 15.73 ms), Cz (M = 342.30 ms, SD = 18.59 ms), and Pz (M = 338.23 ms, SD = 21.35 ms). In contrast, the Go-P3 latency did not change after manipulation of mental fatigue for the control group at three electrode sites. Additionally, the Go-P3 latency significantly increased at Fz (*F*(1, 58) = 14.692, *p* < 0.001, λ = 0.798), Cz (*F*(1, 58) = 5.733, *p* < 0.05, λ = 0.910) and Pz (*F*(1, 58) = 10.370, *p* < .01, λ = 0.848) after manipulation of mental fatigue. On the other hand, there was no significant interaction or main effect for the measurement of the Go-P3 amplitude.

#### NoGo condition

**Fig 7:**
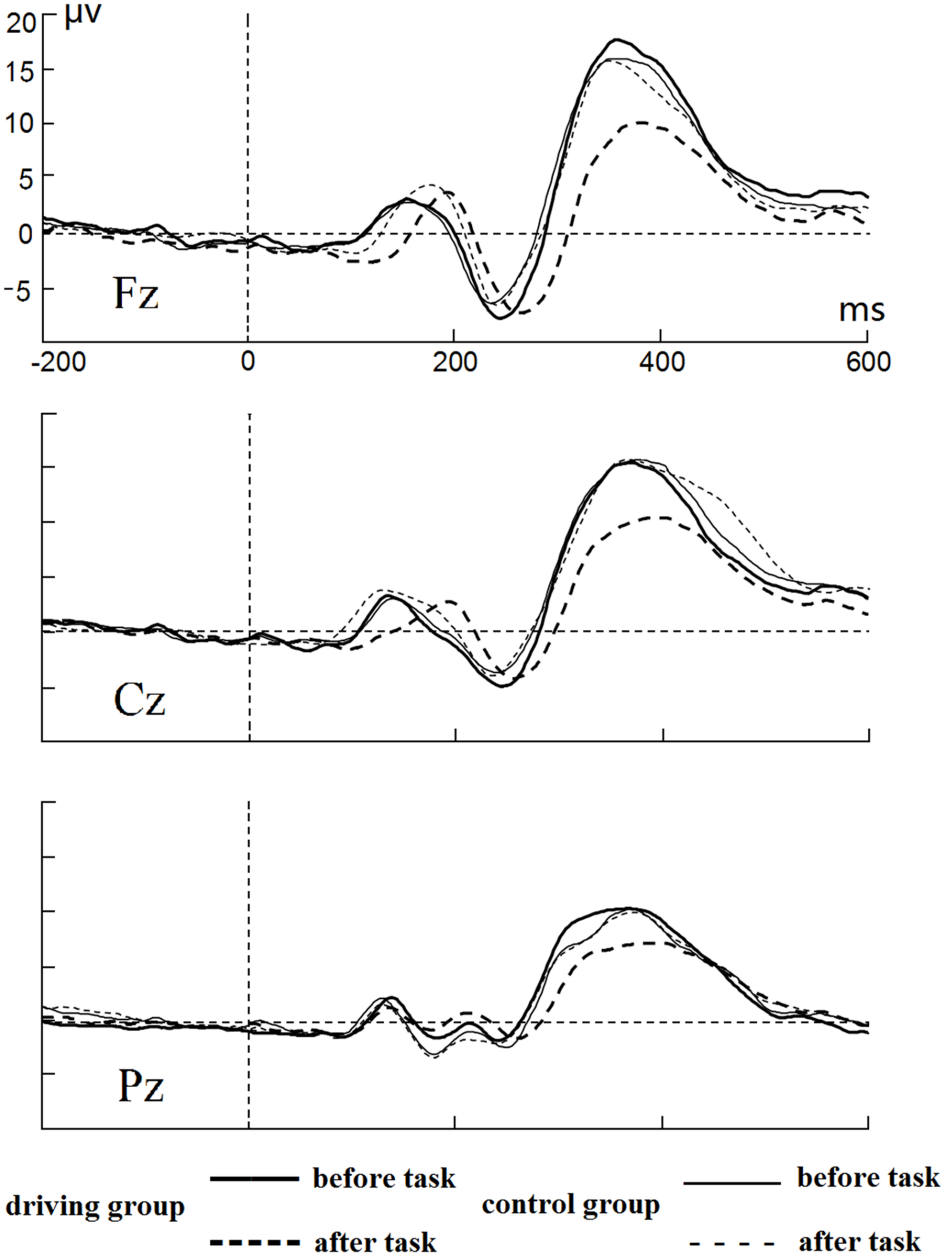
Average ERPs at Fz, Cz and Pz sites for NoGo trails.

As shown in Fig 7, N2 component was successfully elicited in the NoGo trials. The interaction between session and group was significant at Fz (*F*(1, 58) = 5.781, *p* < 0.05, λ = 0.909) and Cz (*F*(1, 58) = 16.108, *p* < 0.001, λ = 0.763) for the NoGo-N2 latency. Simple effects analysis showed that the NoGo-N2 latency significantly increased after manipulation of mental fatigue in the driving group (*ps* < 0.001) at Fz (M = 266.20 ms, SD = 20.36 ms) and Cz (M = 258.93 ms, SD = 20.33 ms), but it did not increase in the control group. Additionally, the main effect of the session was significant for the NoGo-N2 latency, which significantly increased at Fz (*F*(1, 58) = 12.360, *p* < 0.01, λ = 0.824) and Cz (*F*(1, 58) = 8.031, *p* < 0.01, λ = 0.878) after manipulation of mental fatigue. On the other hand, there was no significant interaction or main effect for the measurement of the NoGo-N2 amplitude.

The P3 component was also elicited in the NoGo trials. The session × group interaction was significant for the NoGo-P3 latency at the Fz site (*F*(1, 58) = 21.238, *p* < 0.001, λ = 0.732), Cz (*F*(1, 58) = 19.966, *p* < 0.001, λ = 0.744), and Pz (*F*(1, 58) = 17.829, *p* < 0.001, λ = 0.765). Simple effects analysis indicated that the NoGo-P3 latency significantly increased after manipulation of mental fatigue in the driving group (*ps* < 0.001) at Fz (M = 378.17 ms, SD = 22.95 ms), Cz (M = 389.17 ms, SD = 23.44 ms), and Pz (M = 384.23 ms, SD = 23.00 ms), but it did not increase in the control group. In addition, the NoGo-P3 latency significantly increased after manipulation of mental fatigue at the Fz site (*F*(1, 58) = 11.888, *p* < 0.01, λ = 0.830), Cz (*F*(1, 58) = 11.271, *p* < 0.01, λ = 0.837), and Pz (*F*(1, 58) = 28.901, *p* < 0.001, λ = 0.667). On the other hand, the session × group interactions were significant for the NoGo-P3 amplitude at Fz (*F*(1, 58) = 26.168, *p* < 0.001, λ = 0.689), Cz (*F*(1, 58) = 13.667, *p* < 0.001, η2 = 0.809), and Pz (*F*(1, 58) = 5.948, *p* < 0.05, λ = 0.907). Simple effect analyses showed that after manipulation of mental fatigue, the NoGo-P3 amplitude significantly decreased at three electrode sites in the driving group (*ps* < 0.001), including Fz (M = 10.83 μv, SD = 3.93 μv), Cz (M = 10.62 μv, SD = 3.93 μv), and Pz (M = 7.48 μv, SD = 4.30 μv), but it did not decrease in the control group. The main effect of the session was also significant for this measure. The NoGo-P3 amplitude significantly decreased at Fz (*F*(1, 58) = 31.618, *p* < 0.001, λ = 0.647), Cz (*F* (1, 58) = 17.544, *p* < 0.001, λ = 0.768) and Pz (*F* (1, 58) = 11.068, *p* < 0.01, λ = 0.840) after manipulation of mental fatigue.

### Correlation analysis

**Fig 8:**
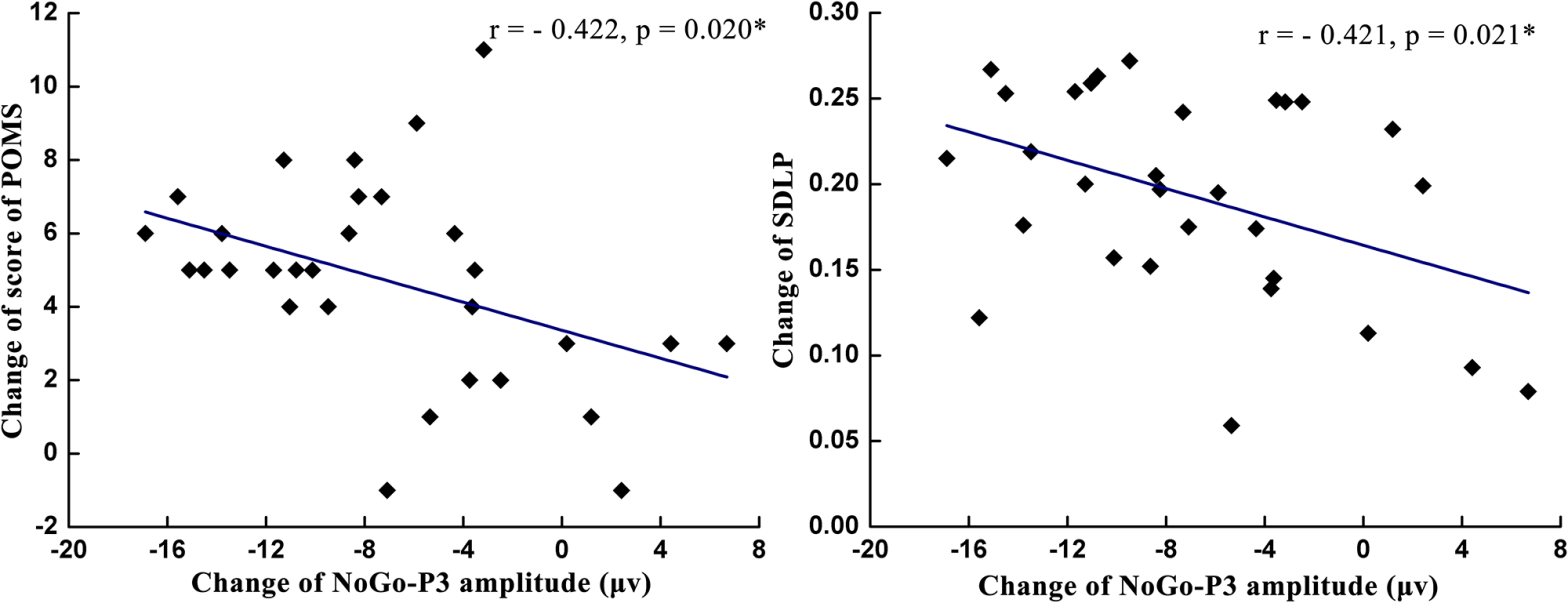
The correlation analysis between NoGo-P3 amplitude changes at the Fz site and the changes of two mental fatigue indices: POMS scores (left) and SDLP (right).

**Fig 9:**
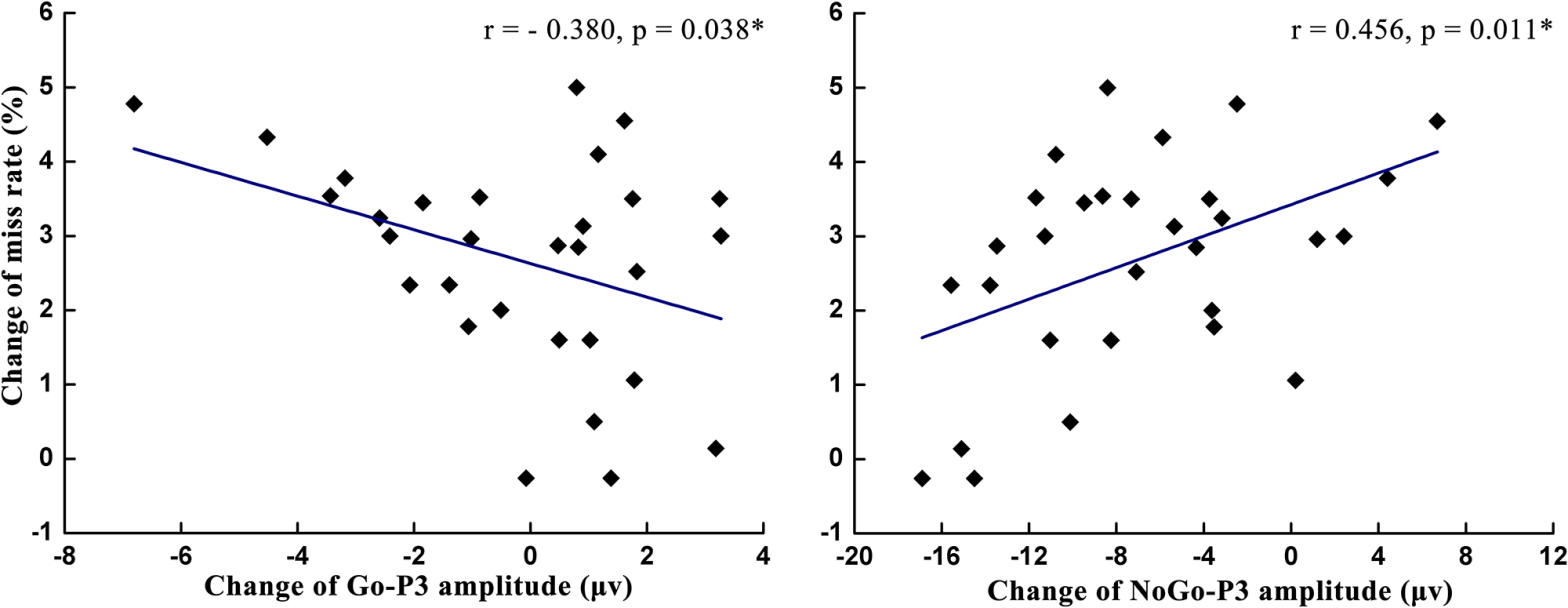
The correlation analysis between the changes of miss rate and Go-P3 amplitude changes (left) and NoGo-P3 amplitude changes (right) at the Fz site.

**Fig 10:**
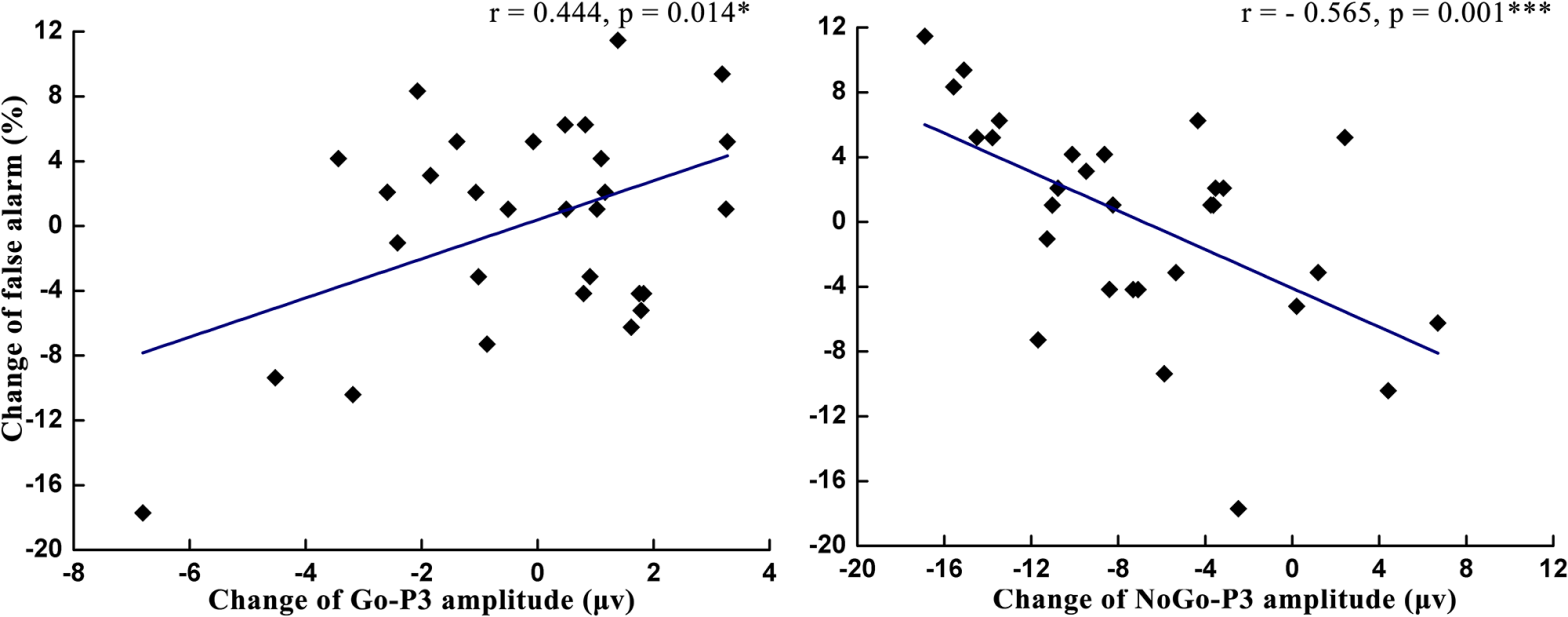
The correlation analysis between the changes of false alarm and Go-P3 amplitude changes (left) and NoGo-P3 amplitude changes (right) at the Fz site.

As shown in Table 1 and Figs 8–10, the change of the NoGo-P3 amplitude was significantly positively correlated with the miss rate change (*r* = 0.456, *p* = 0.011) and was significantly negatively correlated with the change of the POMS score (*r* = -0.422, *p* = 0.020), the SDLP change (*r* = -0.421, *p* = 0.021), and the false alarm change (*r* = -0.565, *p* = 0.001). The change of the Go-P3 amplitude was significantly negatively correlated with the miss rate change (*r* = -0.380, *p* = 0.038) and was significantly positively correlated with the false alarm change (*r* = 0.444, *p* = 0.014). The analysis of correlation indicated that the change of miss rate decreased with the increase of Go-P3 amplitude change and the decrease of NoGo-P3 amplitude change. In contrast, the change of false alarm rate increased with the increase of Go-P3 amplitude change and the decrease of NoGo-P3 amplitude change.

**Table 1:** The correlation between ERP changes at the Fz site and the changes of mental fatigue indices and behavior performance changes (n = 30, participants in the driving group).

		Changes of mental fatigue indices			Behavior performance changes		
		*POMS*	*SDLP*	*PERCLOS*	*RT*	*Miss rate*	*FA rate*
Go-P3 amplitude	*r*	-.315	-.094	-.174	.234	-.380*	.444*
	*p*	.090	.622	.357	.213	.038	.014
Go-P3 latency	*r*	.092	.168	-.101	-.104	.179	-.156
	*p*	.629	.374	.595	.584	.344	.412
NoGo-N2 amplitude	*r*	-.003	-.107	.066	.235	-.111	.247
	*p*	.989	.573	.729	.212	.558	.188
NoGo-N2 latency	*r*	.309	-.018	-.354	-.183	.184	.024
	*p*	.097	.926	.055	.333	.332	.902
NoGo-P3 amplitude	*r*	-.422*	-.421*	.238	.049	.456*	-.565***
	*p*	.020	.021	.206	.796	.011	.001
NoGo-P3 latency	*r*	.144	-.032	.114	.070	-.111	.096
	*p*	.448	.869	.548	.712	.559	.613

*p < 0.05,

**p < 0.01,

***p < 0.001.

## Discussion

This study designed a simulated driving task to induce mental fatigue and examined the impairing effects of mental fatigue on response inhibition in a visual Go/NoGo task. Participants in the driving group reported higher levels of mental fatigue after a 90-min simulated driving task, whereas individuals in the control group reported the same level of mental fatigue after a 90-min watching task. Additionally, participants in the driving group had a higher percentage of eye closure and larger lateral deviations from their lane positions in the first 30 mins compared to the last 30 mins of the 90-min simulated driving task. These results indicated there was effective manipulation of mental fatigue through a prolonged simulated driving task.

We observed increased reaction time and miss rates after manipulation of mental fatigue in the driving group, but this was not the case in the control group. The percentage of false alarms did not change after manipulation of mental fatigue in two groups. The difference between miss rate and false alarm rate may result from the variation of response bias due to mental fatigue, i.e., the rigor of the judgment standard that participants held in the task [40], [41]. According to the signal detection theory, a participant who always says No will never commit a false alarm. In contrast, a participant who always says Yes will never commit a miss. In this study, mental fatigue led to a decline in the task performance, however, participants in the driving group might become more conservative to perform a Go/NoGo task. This conservative strategy made a participant in the driving group more likely to provide the response No to avoid a false alarm, which resulted in the same level of false alarms after manipulation of mental fatigue.

The latency of NoGo-N2 was delayed after participants in the driving group performed a 90-min simulated driving task, but this trend was not similar for the amplitude of NoGo-N2, which was consistent with the findings of Kato et al [14]. According to previous studies, the NoGo-N2 effect represented the earlier stages of response inhibition, i.e., the conflict detection process [21, 23, 24, 42]. The delayed latency of N2 in the present study might suggest that mental fatigue influences the time course of inhibitory activity by slowing down the speed of response inhibition. More specifically, mental fatigue attenuated the speed of conflict detection between the internal representations of the Go and NoGo stimuli. The NoGo-N2 amplitude did not change after manipulation of mental fatigue, which was consistent with the stable false alarm rates. The amplitude of the NoGo-N2 component represents the intensity of conflicts between trials and is sensitive to exogenous factors, such as stimulus features, stimulus presentation mode and stimulus ratio [23, 24]. In the current study, these factors were controlled at the same level before and after the manipulation of mental fatigue. Participants may perceive the same intensity of conflicts between trials in two Go/NoGo tasks, leading to the same amplitude of the NoGo-N2 component between the pre- and post-test.

The latency of Go-P3 was also delayed after participants in the driving group performed a 90-min simulated driving task. Since P3 latency reflects the speed of high-level cognitive activity [29], the increased Go-P3 latency suggests that mental fatigue delayed the speed of information processing during the stimulus-evaluation and decision-making phases of response inhibition [14]. Based on this finding, increased reaction times on Go trials after the manipulation of mental fatigue may be caused by the delayed stimuli evaluation time. In addition, we found that mental fatigue did not affect the Go-P3 amplitude, but the NoGo-P3 amplitude decreased after participants in the driving group performed a 90-min simulated driving task. The P3 amplitude serves as an indicator of cognitive resources [27–29, 43]. In the current study, the stable Go-P3 amplitude showed that attentional resources devoted to Go stimuli remained the same after manipulation of mental fatigue. The decreased NoGo-P3 amplitude suggested that participants experienced increased difficulty in allocating cognitive resources to NoGo stimuli. These changes in the Go-P3 and NoGo-P3 amplitude were consistent with the results of Kato et al [14] but were inconsistent with Guo et al. [15]. The inconsistency between the results of Guo et al and our results may be caused by different settings of stimulus ratios. Stimulus ratios change a participant’s expectation to stimuli [44, 45], and such expectations lead to biased attention to resource allocation, which eventually changes the P3 amplitude accordingly [46]. In the study by Guo et al, the probability of NoGo trials (50%) was equal to the probability of Go trials (50%). Participants allocated the same amount of attentional resources to both stimuli. In contrast, the probability of Go trials (80%) was higher than NoGo trials (20%) in the study by Kato et al and our studies. Participants expected that a Go stimulus was more likely to appear in each trial and paid more attention to a Go stimulus. As a result, the amplitude of Go-P3 was not affected by mental fatigue, whereas the amplitude of NoGo-P3 decreased due to fewer available attentional resources due to mental fatigue. The correlation analyses also supported this explanation. We observed that the changes of miss rate decreased with the increase of Go-P3 amplitude changes, while the changes of false alarm rate increased with the decrease of NoGo-P3 amplitude changes at the Fz site. After the manipulation of mental fatigue, the miss rate increased but Go-P3 amplitude did not decrease, the false alarm rate was the same but the NoGo-P3 amplitude decreased. There findings indicated that under the influence of mental fatigue, participants in the driving group devoted less attention resource to NoGo stimuli but stable or even more attention resource to Go stimuli to maintain the primary task performance.

The induction of mental fatigue and the assessment of response inhibition in the previous studies [13–15] were investigated based on traditional psychological cognitive tasks, e.g., a continuous Go/NoGo task. However, the fatigue level and operation performance are modulated by the characteristics of tasks performed [34]; therefore, mental fatigue induced by cognitive tasks in the laboratory environment made their conclusions difficult to apply to real-life situations. Additionally, the previous studies did not use a control group to justify whether a continuous Go/NoGo task successfully induced mental fatigue or determine which component of ERP was affected by mental fatigue. In comparison, mental fatigue was induced by a 90-min simulated driving task with higher ecological validity in this study, and another 90-min watching task was used for comparison. Further investigation could include a response inhibition task in a simulated driving task [47, 48] and compare the effects of mental fatigue on the behavioral patterns and brain mechanisms between a traditional cognitive task and a simulated driving task.

In the current study, we examined only the effects of mental fatigue on response inhibition during a visual Go/NoGo task. Whether the results from a visual Go/NoGo task are valid for other perceptual modalities (e.g., an auditory Go/NoGo task) needs further investigation.

## Conclusion

The present study demonstrated that mental fatigue deteriorated a participant’s response inhibition in a visual Go/NoGo task, which was reflected by prolonged reaction time, increased miss rates, and delayed latency for NoGo-N2 and Go-P3 and decreased amplitude for NoGo-P3. These results showed that mental fatigue not only led to fewer attentional resources allocated to the NoGo stimuli but also delayed the speed of response inhibition. These findings revealed the underlying neurological mechanisms about the impairing effects of mental fatigue on response inhibition.
